# The Effects of Progesterone Therapy on the Gestation Length and Reduction of Neonatal Complications in Patients who had Received Tocolytic Therapy for Acute Phase of Preterm Labor

**DOI:** 10.5812/ircmj.7947

**Published:** 2013-10-05

**Authors:** Marzie Lotfalizadeh, Nayereh Ghomian, Amirreza Reyhani

**Affiliations:** 1 Women Health Research Center, Department of Gynecology and Obstetrics, Faculty of Medicine, Mashhad University of Medical Sciences, Mashhad, IR Iran

**Keywords:** Premature Birth, Progesterone, Gestational Age, Tocolysis, Hydroxyprogesterones, Apgar Score

## Abstract

**Background:**

While tocolytic therapy can halt the process of delivery, some patients return before the 37th week of pregnancy with recurrence of preterm labor signs.

**Objectives:**

This study was designed to evaluate the efficacy of progesterone in the prolonging of gestation and reduction of neonatal complications.

**Material and Methods:**

In a clinical trial in 2010, 110 singleton pregnant women admitted at Imam Reza Hospital, Mashhad, Iran, with the diagnosis of preterm labor were divided into three groups: 400 mg/d vaginal progesterone suppositories; 250 mg/w 17-alpha-hydroxyl-progestrone-caproate; and a control group with no additional treatment. After delivery, we assessed the duration between the first phases of labor to the recurrence of preterm labor. The neonatal complications, apgar score, birth weight, need for admission to NICU, and congenital malformations were compared between groups.

**Results:**

The mean gestational age was 34± 3 weeks in the first, 33.5 ± 3 weeks in the second and 32.5 ± 2 weeks in the control group. The duration of first phase of labor was 31 ± 17 days in the first, 36 ± 14 days in the second and 26 ± 22 days in the control group. The difference between study groups and the control group was significant (P < 0.005). The complications were lower in progesterone-receiving group in comparison to the control group.

**Discussion:**

This study reveals that progesterone can significant reduce the rate of recurrent preterm labor and the several possible neonatal complications among women who had treated with tocolytics to suppress the acute phase.

## 1. Background

Prematurity is considered as one of the major etiologies of neonatal mortality. (1, 2). Currently, tocolytic therapy by beta-adrenergic agonists, calcium-channel-blockers, and oxytocin antagonistsis and progesterone for prevention of preterm labor is considered the main strategy to solve this issue (3, 4). These medicines are used to target the responsible stimuli for induction of delivery. While they are not clearly identified, several mechanisms such as change in the amniotic fluid eicosanoid concentrations, (5) amniotic fluid cell death nucleosomes' levels, (6) inflammation and subsequent alteration in the concentration of cytokines, (7) systemic or local alteration in levels of steroidal hormones, (8, 9) and cervical length, (10) has been proposed. It is demonstrated that amniotic fluid F2-isoprostane, PGE2, and PGD2 have higher concentrations at term while in preterm births, there is upper level of PGF2α. (5). Furthermore, Soloff showed that progesterone can reduce uterine excitability by affecting the expression of calcium and voltage-operated K(+) channels and down-regulation of receptors involved in myometrial contraction and the proteins involved in the cross-linking of actin and myosin filaments to produce uterine contractions (9). In addition to prevention from delivery, pre-labour progesterone has now several other indications such as in threatened abortion, supporting luteal phase in IVF and management of preterm labour. (11-13). Another indication for progesterone in pregnancy is the maintenance therapy in preterm labour (14). While tocolytic therapy can halt the process of delivery, (15-17) some patients return before the 37th week of pregnancy with recurrence of preterm labour signs. Those above 34th week do not need tocolytic therapy any more, but as it is still preterm situation, the neonates might need to be admitted at NICU and the risk of complications still persists (18). Therefore, to prevent recurrence, increasing the length of pregnancy and acquiring better birth weight, some patients are treated with oral tocolytics as a maintenance therapy (19); however, this strategy is controversial and some other medications such as Atosiban and progesterone have been suggested for the maintenance therapy (14, 20). There are some evidences that progesterone can be used in this phase. Intra-muscular (IM) weekly injection of 17-alpha-hydroxyl-progestrone-caproate is an appropriate medication for those at risk of preterm labour. (21). This important clinical trial shows that in comparison to women receiving placebo, they had lower risk of preterm labour and perinatal mortality. The efficacy of vaginal progesterone suppositories in lowering the rate of preterm labour has also been reported. (22). However, there are studies that explain the lack of effectiveness of progesterone in prolonging pregnancy (23, 24).

## 2. Objectives 

Our study was designed to evaluate the efficacy of progesterone maintenance therapy in the patients who had received tocolytic therapy for preterm labour.

## 3. Patients and Methods

In a clinical trial in 2010, singleton pregnant women admitted at Imam Reza Hospital, Mashhad, Iran, with the diagnosis of preterm labour were assessed regarding length of gestation and neonatal complications following progesterone maintenance therapy. The diagnosis was based on the presence of at least four contractions per minute, accompany to 2 cm dilatation, or 80% effacement, or progressive cervical changes (dilatation of 1 cm per hour). Inclusion criteria were gestational age of 26 to 36 weeks (according to an accurate LMP or first-trimester ultrasound), singleton pregnancy, premature contraction and cervical changes with intact amniotic membrane. Exclusion criteria were chorioamnionitis, fever, evidence of IUGR, oligohydroaminos, fetal abnormalities, gestational diabetes and hypertension or any other maternal or fetal medical complications, any contraindication for tocolytic therapy, dilatation 4 cm or more, progression towards delivery despite of treatment with tocolytic and any indication for urgent termination of pregnancy by cesarean section. All the patients referring to the hospital with initial diagnosis of preterm labour were first rehydrated by Ringer Lactate 1000 cc in an hour and received analgesic (50 mg pethidine IV). If the contractions were not true and became suppressed, there was no need for additional treatments and patients were excluded from the study. Otherwise, magnesium sulfate with a loading dose of 4 g IV and maintenance dose of 1 - 2g per hour or nifedipine one tablet each 20 minutes up to three doses as a loading dose and one tablet each six hours up to 24 hours as a maintenance dose was started. Twelve hours after cessation of contractions, we stopped tocolytic therapy. In addition, all patients received betametasone 12 mg/day IM up to two doses and ampicillin 1 g/6h up to 48 hours and then oral amoxicillin 500 mg/6h and erythromycin 400 mg/6h for 5 days. After the control of premature contractions and 24 hours follow-up, information about the maintenance therapy and the aims and process of the research was given to the patients. Signing a written consent according to the guidelines of the ethics committee of Mashhad University of Medical Sciences, volunteers were divided into three groups: The first received 400 mg/d vaginal progesterone suppositories until the 37th gestational week; in the second group, 250 mg/w 17-alpha-hydroxyl-progestrone-caproate was given until the 37th gestational week; the third as a control group received no additional treatment. All the patients were controlled weekly in the high-risk obstetrics clinic of Imam Reza, Mashhad University of Medical Sciences. If any sign of preterm labour was observed, a vaginal examination was performed by the obstetrics and if the initiation of labour was approved, we admitted the patient for possible evaluation of any indication for termination. After delivery, we assessed neonatal complications, apgar score, birth weight, need for admission to NICU, and congenital genitourinary and musculoskeletal malformations and compared them between the study groups. In addition, the duration between the first phases of labor to the recurrence of preterm labor was considered as the prognosis of each treatment modality for prevention of preterm labor.

## 4. Results

From 233 pregnant women admitting to the hospital for preterm labor, only 110 agreed and fulfilled the criteria to continue our study. From these cases, 37 accepted the treatment with 400 mg/d vaginal progesterone suppositories, 37 received 250 mg/w 17-alpha-hydroxyl-progestrone-caproate and 36 were arrived in the control group. There was no significant difference between these groups regarding the mean age of mothers. The mean gestational age at the time of labor was 34 ± 3 weeks in the first, 33.5 ± 3 weeks in the second and 32.5 ± 2 weeks in the control group. This difference was not significant among the groups (P > 0.05). The duration of first phase of labour was 31 ± 17 days in the first, 36 ± 14 days in the second and 26 ± 22 days in the control group. There was no difference between the first and second groups; however, the difference between study groups and the control group was significant (P < 0.005). The percentages of perinatal complications including low birth weight (< 2500 g), NICU admissions and neonatal infections were lower in progesterone-receiving groups in comparison to the control group (27% vs. 50%, 24.5% vs. 38.5% and 3% vs. 8.5%, respectively) (Table 1). There was no case of congenital genitourinary and musculoskeletal malformations in the neonates of each group and none of the mothers receiving progesterone complained from common hormonal complications such as headache, fluid congestion or mood change. 

**Table 1.** The Perinatal Complications Among Therapeutic Group

Perinatal Complications	Case Groups		The mean between Two Case Groups	Control group	P value
	First	Second			
**Low birth weight (< 2500 g), No., (%)**	10 (27	10 (27)	20 (27)	18 (50)	0.02
**NICU admissions, No., (%)**	10 (27)	8 (21.5)	18 (24.5)	14 (38.5)	0.11
**Neonatal infections, No., (%)**	1 (3)	1 (3)	2 (3)	3 (8.5)	0.21
**Duration of first phase of labor, Mean ± SD**	31 ± 17	36 ± 14	33.5 ± 15	26 ± 22	< 0.01

## 5. Discussion

The incidence of preterm labor has increased globally and the management of this complication is still challenging (14, 15, 23, 25-27). Even for the treatment of acute phase, some insist that beta-adrenergic agonists, calcium-channel-blockers, and oxytocin antagonists are the first-line medications (25) while others believe that the only effective tocolytic drugs are the prostaglandin inhibitors and magnesium sulfate should not be used to treat premature labor (26). In this clinical trial, efficacy of progesterone maintenance therapy after treatment with tocolytics for preterm labor was assessed and approved. It seems that it can preserve the relaxation made by the initial tocolytic therapy for the acute phase of preterm labor. Thus, a considerable reduction in the complications such as low birth weight, NICU admissions, and neonatal infection is achievable, as it is shown that the length of gestation has a significant impact on the neonatal complications and mortality (28). Some studies support the findings of our study (29-32). For instance, it is shown that 17-alpha-hydroxyl-progestrone-caproate when prescribed as weekly injections for those at risk of preterm labor can prolong pregnancy. So the patients had lower risk of preterm labor and perinatal mortality (21). Facchinetti showed that it can reduce the incidence both in nuli- and pluriparous women with a previous history of preterm labor (29). Data about the efficacy of vaginal progesterone suppositories in lowering the rate of preterm labor has been previously reported. In this study, prophylactic application of vaginal progesterone suppositories in women significantly reduced the risk of preterm labor and perinatal mortality (22). In addition, the risk of neuromuscular, limb, genitourinary and gastrointestinal abnormalities is not higher in those treated with progesterone in the late pregnancy comparing to the control group. However, there are still some controversies regarding the optimal formulation, route of administration, dose, and gestational age at initiation (24, 31). It should also be noticed that the effects of progesterone are related to the vehicle used and the route and time of administration (33). As mentioned above, the main routes are intra-muscular and suppository, whilst the oral route seems to be non-beneficial (23). However, an important study has revealed that women receiving 17-alpha-hydroxyl-progestrone-caproate prophylaxis before 27 weeks' gestation might have even an increased risk for preterm labor (34). Furthermore, it should be kept in mind that progesterone has no role in the prevention of preterm labor in twin pregnancy (35, 36) weekly injections of 17-alpha-hydroxyl-progestrone-caproate as well as vaginal progesterone suppositories can lead to a significant reduction in the rate of recurrent preterm labor among women who had treated with tocolytics to suppress the acute phase and also there is a considerable reduction in the neonatal complications. However, it seems that further future studies are needed to accurately define the mechanism of action of progesterone drugs in the prevention of preterm labor and to establish the optimal formulation, route of administration, dose, and gestational age at initiation.
